# Comparative Analysis of Preference in Contemporary and Earlier Texts Using Entropy Measures

**DOI:** 10.3390/e25030486

**Published:** 2023-03-10

**Authors:** Mahdi Mohseni, Christoph Redies, Volker Gast

**Affiliations:** 1 Department of English and American Studies, University of Jena, 07743 Jena, Germany; volker.gast@uni-jena.de; 2 Experimental Aesthetics Group, Institute of Anatomy I, Jena University Hospital, University of Jena, 07740 Jena, Germany; christoph.redies@med.uni-jena.de

**Keywords:** Approximate Entropy, Shannon Entropy, fictional texts, non-fictional texts, canonical texts, non-canonical texts, contemporary texts, bestseller books, POS tags, text classification

## Abstract

Research in computational textual aesthetics has shown that there are textual correlates of preference in prose texts. The present study investigates whether textual correlates of preference vary across different time periods (contemporary texts versus texts from the 19th and early 20th centuries). Preference is operationalized in different ways for the two periods, in terms of canonization for the earlier texts, and through sales figures for the contemporary texts. As potential textual correlates of preference, we measure degrees of (un)predictability in the distributions of two types of low-level observables, parts of speech and sentence length. Specifically, we calculate two entropy measures, Shannon Entropy as a global measure of unpredictability, and Approximate Entropy as a local measure of surprise (unpredictability in a specific context). Preferred texts from both periods (contemporary bestsellers and canonical earlier texts) are characterized by higher degrees of unpredictability. However, unlike canonicity in the earlier texts, sales figures in contemporary texts are reflected in global (text-level) distributions only (as measured with Shannon Entropy), while surprise in local distributions (as measured with Approximate Entropy) does not have an additional discriminating effect. Our findings thus suggest that there are both time-invariant correlates of preference, and period-specific correlates.

## 1. Introduction

What makes a text “successful”, in the sense that it sells well, reaches a broad readership and/or acquires prestige among educated readers and critics? Is it promotion, network effects, economic or social circumstances—or perhaps the “quality” of the text itself? These questions have recently been addressed in a variety of studies in the field of computational aesthetics, aiming to identify observable correlates of preference in the structure of a text [1,2,3,4,5,6,7]. In empirical aesthetics the term “preference” is used to capture aesthetic attitudes towards cultural artefacts [8]. Such attitudes can be held both at an individual level—specific readers enjoy specific (types of) books—and at a community level—specific types of texts, and their authors, may obtain recognition and acquire prestige [9,10,11].

On the assumption that aesthetic experience can have a foundation in the cultural artifact itself, a natural question to ask is whether, or to what extent, correlations between properties of a work of art, such as a literary text, and the aesthetic response in readers, are invariant across time, space, and cultural environments, or whether they are dependent on such variables. In the present study we address this question by studying correlations between structural properties of texts and degrees of (community-level) preference across two time periods. Specifically, the central question is to what extent the textual determinants of preference in the 19th and early 20th centuries were the same as, or different from, the textual determinants of preference today. As we operationalize preference differently for the two time periods (canonization and sales figures), the notion of ‘preference’ itself, in the context of prose texts, is under scrutiny as well.

The study of correlations between measurable properties of cultural artefacts on the one hand, and preference on the other, is obviously non-trivial. There are two major challenges: first, preference for texts is not immediately measurable, as it is for, say, visual stimuli, where preference for large numbers of images can be recorded directly and in real time [12]. We thus need to operationalize that concept in a reasonable (valid) way. Second, we do not know at present what types of structural properties will show correlations with our operationalizations of preference. The exploratory nature of this study (as well as other studies carried out in this spirit) should thus be obvious. Correspondingly, even minor correlations (small effect sizes) are of interest to us, as long as they are statistically significant.

In the present study we operationalize preference in terms of reception or, put differently, the scope of the readership. We deal with texts from two time periods: with the contemporary period, spanning the time between 2000 and 2020, and from an earlier period, covering the time between 1813 and 1922. For each period we (necessarily) use different operationalizations of preference: The earlier texts are divided into canonical and non-canonical texts, using the canon of Western literature [13,14] as a criterion of classification (see also [7,15]). Canonical texts form part of the cultural backbone and historical memory of a society [16]. They are often time-honoured and are included in syllabuses at schools and universities. Given their prestige and institutional support, they reach a broad readership distributed over a large time span.

The reception of contemporary texts cannot be measured via canonization, which is a process that takes some time and involves several stakeholders, such as publishing houses, academics and government departments. These texts are therefore classified according to their commercial success, and thus divided into bestsellers and non-bestsellers (see also [17,18,19]). Like canonical texts, bestsellers have reached a broad readership. This readership is not distributed over time, however, but constituted by a single ‘cohort’ at the time of publication. Obviously, the two operationalizations of preference (canonization and sales figures) are not identical. What they share is that they measure reception; they (may) differ in the type of readership. In interpreting our results, that difference of course needs to be taken into consideration.

As for the structural properties of texts that are potential correlates of preference, there are two central challenges. The first question is what type of observable properties we measure. We assume that the aesthetic experience in reading is a function of both what a text is about—for instance, the plot and the characters in a novel—and how it is written. Figures may be characterized in a specific way (explicit vs. implicit characterization) [20], and the state of affairs can be described by the narrative voice, through dialogues or interior monologue, etc. [21]. While such elements of style are hard to measure directly, they have structural reflexes in texts, for instance, insofar as they imply the use of different discourse modes [22] which, in turn, come with different distributions of parts of speech [23,24]. For example, the mode of narration typically implies the use of past tense verbs, dialogue comes with a high proportion of pronouns and verbs, description requires adjectives, etc. Given the association between register, style, and discourse modes and the distribution of parts of speech [23,24,25,26], the latter category, which is observable and measurable, figures prominently in our work.

The second major challenge of our work concerns the type of statistic that may be informative with respect to the degree of preference. Previous studies, inspired by research in the domain of vision (for a review, see [12]), have focused on global statistical properties such as the variability (of observables) in a text [15,27,28], long-range correlations [6,15,29] and various indicators of predictability or surprise [4,7]. In the present study we use two measures of surprise—Shannon Entropy and Approximate Entropy—as the aesthetic experience has been shown in previous work to be driven by the interplay between expectation and surprise [7,30,31] (moreover, see [32,33] for a discussion on “unification” and “diversification” in text).

Specifically, the present study investigates the differences and similarities in the relationship between the degree of surprise in the textual structure and (community-level) preference, in two time periods, the 19th and early 20th centuries, and the contemporary period. Preference is operationalized as canonization for the earlier texts, and in terms of sales figures for the contemporary texts. Similarities can be expected on the assumption that certain determinants of preference are time-invariant (universal), and do not vary significantly with the readership. Differences can be expected because writing styles are known to vary from one period to the next [25], and because literature is embedded into socio-cultural contexts, with changing aesthetic preferences in all domains of culture (music, painting, architecture, etc.). Moreover, the two operationalizations of preference can be expected to have different types of reflexes.

As reviewed in more detail in [7], preference has been operationalized in terms of the scope of the readership in previous studies under different terms, such as “success” [27], “popularity” [34], being “professional” vs. “amateur” [1], or “information-based energy” [35]. Data from websites and social networks have been used in some previous studies to model readers’ preference, for instance, the download counts from the website of the Gutenberg Project [2], or ratings of readers on the website Goodreads [3,5].

Some previous studies have referred to the Nobel Prize as a gauge for high quality or success. For example, Febres and Jaffe [4] analysed the categories of Nobel laureates and non-Nobel laureates in two languages, English and Spanish, using global properties, such as entropy, lexical diversity and word frequency distribution in texts. Their results showed that statistical measures can be predictive of the category of texts, with a higher performance for Spanish compared to English texts. Bizzoni et al. [6] classified Nobel prize winners from other texts using the fractality of sentiment arcs. They showed that the distribution of self-similarity measures in the two text categories under analysis differed, and that the degree of fractality of higher-quality texts is likely to be located in a specific range of values.

Mohseni et al. [15] approached the discrimination of canonical from non-canonical texts using textual properties of texts represented in the form of a series. They used sentence length, the frequencies of POS tags per sentence, the lexical diversity metric MTLD, and topic probabilities to numerically represent the structure of a text, and determined the variance and long-range correlations of the series corresponding to the texts. Training a classifier with the calculated values, they were able to distinguish fictional from non-fictional and, within the fictional category, canonical vs. non-canonical English texts with acceptable accuracy.

Success has also been defined based on sales figures. Yucesoy et al. [17] and Wang et al. [18] analysed texts in the New York Times Bestseller lists and Vasyliuk et al. [19] investigated bestseller books on Amazon. However, they did not analyse the texts of books, but rather restricted their analyses to more straightforward statistical information and metadata, such as the time of publication, number of reviews, genre, and price, and related the success of the texts to non-textual factors.

In the present study we adopt the approach to textual aesthetics proposed by Mohseni et al. [7,15]. We assume that a pleasant reading experience emerges from an interplay of predictability and surprise. Previous work has shown that canonical literature differs from non-canonical literature in its degree of predictability. Mohseni et al. [7] analysed two types of series derived from texts, sequences of sentence lengths and of frequencies of part-of-speech (POS) tags in fixed-size windows of text (see Section 2.3). Two entropic metrics were computed, Shannon Entropy (ShEn) and Approximate Entropy (ApEn), for the distributions of relevant text properties. ShEn measures (ir)regularity as a global structural property. ApEn determines (un)predictability as a sequential characteristic of underlying text property series (see Section 2.4). This method was also applied in the present study, with a different dataset. Note that the present study primarily focuses on the classification of texts on the basis of preference levels. The temporal dimension comes into play insofar as we compare texts of preference levels from two periods of time. We do not perform temporal classification, in the sense that the time of writing is the category of classification. Approaches to temporal classification are nevertheless summarized in the Supplementary Materials, Section S1.

The paper is organized into three sections. Section 2 contains a description of the data and methods. Section 3 presents the results, which are discussed in Section 4.

## 2. Data and Methods

### 2.1. The Jena Corpus of Expository and Fictional Prose

The present study is based on the Jena Corpus of Expository and Fictional Prose (JEFP), version 2.0. The corpus was compiled for a comparison of different types of fictional and non-fictional texts from the 19th and early 20th centuries, here called “earlier” texts, and it has been used for the study of questions relating to empirical aesthetics [7,15]. The JEFP Corpus comprises three sub-corpora: canonical/fictional, non-canonical/fictional and non-fictional (Table 1). The canonical sub-corpus consists of 76 texts that form part of the Western literature canon Bloom [13]. It represents a collection of fictional texts that are widely known among the educated population, often taught in school and discussed in academic discourse. The category of non-canonical fictional texts comprises 130 texts that were obtained from the Project Gutenberg website. It represents the non-preferred earlier texts. Finally, the sub-corpus of non-fictional texts contains 185 texts from different genres such as architecture, astronomy, geology, geography, philosophy, psychology and sociology. These texts were also obtained from the Project Gutenberg website.

### 2.2. The Jena Corpus of Contemporary Expository and Fictional Prose

For our comparative study, we also needed a corpus of contemporary texts to compare them with the earlier texts in the JEFP corpus. Thus, we compiled a corpus which contained categories analogous to those of the JEFP corpus (preferred/fictional, non-preferred/fictional and non-fictional). We called this corpus the “Jena Corpus of Contemporary Expository and Fictional Prose” (JCEFP).

To compile the list of preferred contemporary texts, we used the New York Times Bestseller list, which is published weekly in the New York Times Book Review. Some books manage to appear on the list for several weeks, and some lose their rank after only one week in competition with other books. We selected ninety-three texts from lists of the New York Times Fiction Best Sellers published from 2000 to 2020. Our selection was based on lists taken from Wikipedia for each year.

To build the category of non-preferred contemporary texts, we used the website www.smashwords.com (accessed on 11 March 2021), which allowed us to search for texts based on various criteria, such as genre, length and price. In this part of the corpus, we only included freely available fictional texts, assuming that texts promising commercial success will not be distributed for free by a publisher. This part of the corpus consequently contains no bestsellers, as bestsellers would have to be bought. For a book to be free does not of course mean that the book is not read by anyone. In fact, free distribution could be an incentive for people interested in popular literature to read the texts. Moreover, if an author manages to publish a successful text later, their previous, less-successful texts may find more readers (as in the case of B. Obama’s first book *Dreams from my Father*, for instance). Still, at the time of publication the texts are clearly non-bestsellers, and books that are not promoted by publishers. The non-preferred sub-corpus thus compiled by us contained 110 texts.

Non-fictional texts were randomly selected from different genres, e.g., philosophy, psychology, sociology and natural science, similar to the genres that we included for texts in the JEFP corpus. The contemporary version of the non-fictional sub-corpus contained 122 texts. Table 2 presents the summary statistics for the JCEFP corpus. As we selected bestselling books from lists from 2000 to 2020, the category of non-preferred non-fictional texts was also restricted to texts that were published after 2000. Table S1 in the Supplementary Materials lists all texts with the metadata.

All texts in both the JEFP corpus and the JCEFP corpus were pre-processed in the same way. We removed the tables of contents and indices and cleaned up the texts partly manually and partly automatically using regular expressions to fix broken lines and hyphenated words.

To segment texts into sentences and to assign POS tags to tokens, we used the Stanza package for Python [36], a neural-based text processing toolbox with high accuracy. We used the toolbox with the default pre-trained model for English (UD English EWT, version 1.0.0 [37]).

Note that previous studies have shown no underperformance of taggers for texts from the 19th century. This is probably due to the fact that orthography was already standardized at that time. For instance, Schneider et al. [38] showed that if a POS tagger was trained on contemporary texts and applied to historical texts written after 1800, the performance would not drop. They also analysed the tagging errors and showed that most POS tagging mistakes were found in lower-level categories within the major classes; for example, between NN (noun, singular or mass) and NNP (proper noun, singular), and between VB (verb, base form) and VBP (verb, non-third person singular present). Such errors would not affect our results because we analysed the distribution of major word categories (see Section 2.3).

### 2.3. Properties Underlying Textual Structure

To analyse the structural organization of texts, we took the same approach as Mohseni et al. [7]. We represented and analysed texts by seven text properties: sentence length and the frequencies of six major parts of speech in fixed-size windows: Noun, Verb, Adjective, Adverb, Pronoun and Preposition. Sentence length was measured as the number of tokens in a sentence, including all words and punctuation marks. Each major part-of-speech (POS) included all relevant sub-categories. For example, plural, singular, common and proper nouns all were counted as Noun. All forms of verbs, base form, past tense, past participle and gerund, were treated similarly as Verb. Adjective and Adverb included simple, comparative and superlative types. Pronoun covered personal and possessive pronouns.

To build series of part-of-speech (POS) tags, we counted the number of each POS tag in subsequent windows of 25 tokens of text. As mentioned in Mohseni et al. [7], the window size does not have a significant effect on the results as long as it is within reasonable limits. By windowing, we split each text into a sequence of fixed-length segments. Fixed-length segmentation eliminates undesirable effects of correlation between sentence length and frequencies of POS tags. Each window of text is called a “box”. Each box is like a small bag of words, in which the internal structure of the texts is ignored and only the frequency of POS tags is determined. We therefore call this approach a ‘sequence of boxes’ approach. If the order of the boxes in the sequence was taken into account, we analysed the underlying structural design of a text (as in the case of Approximate Entropy; Section 2.4). If we ignored the linear order of the boxes, we analysed the global distribution of POS tags in a text (as in the case of Shannon sntropy; Section 2.4).

### 2.4. Approximate Entropy and Shannon Entropy

To measure the degrees of (ir)regularity and (up)predictability in a series of text properties (Section 2.3) we used two entropy measures: Shannon Entropy (ShEn) and Approximate Entropy (ApEn) [39]. ShEn is a measure of global distribution and is computed as

$$-\sum_{x\in S_{x}}p\left(x\right)\log p\left(x\right)$$

where $S_{x}$ is the set of all possible events *x*. ShEn assumes that events happen independent of each other. This metric measures the degree of uncertainty. If the probability of all events is equal, the system has the highest uncertainty, and as a result, ShEn takes its maximum value.

Conversely, ApEn is a measure of sequential organization (cf. Supplementary Materials, Section S2). It was proposed to measure the degree of (ir)regularity in a series according to the distance (dissimilarity) of sub-sequences to each other. As variation is an intrinsic characteristics of a series, in ApEn some level of fluctuation is “tolerated”. If the difference between two sub-sequences lies within the “tolerance” level, it is assumed that “similarity” is not violated. In the computation of ApEn, the sub-sequence matches of length *m* are compared with sub-sequence matches of length m+1. In a sequence with a high level of fluctuation, longer sub-sequences are less-likely to be similar to each other, which in turn leads to a higher ApEn value. In exploratory studies, the parameters of ApEn, i.e., *m* and *r*, are usually set to 2 and 20% of the standard deviation, respectively, (see, for example, [7,40,41,42]).

In our experiments we used both ShEn and ApEn. ShEn measures surprise based on global distributions. AppEn measures surprise based on (ir)regularities in the series. Note that a high degree of AppEn implies a high degree of ShEn but not vice versa. We first calculated the degree of irregularity (or unpredictability) in a series of text properties. On this basis we determined to what extent any observed difference originate from the global distribution of the features (ShEn), or from their sequential organization (ApEn). The code that we used to calculate features is accessible at https://github.com/mohsenim/Surprise (accessed on 5 February 2023).

## 3. Results

Our analyses implied a two-dimensional comparison. We carried out (i) a comparison of preferred and non-preferred fictional texts, for each period, and (ii) a comparison of the differences for each period. We used our two corpora, JEFP and JCEFP, which, as explained in Section 2.1, contained preferred texts (canonical texts in JEFP; bestselling contemporary texts in JCEFP), and non-preferred texts (non-canonical texts in JEFP; non-bestselling contemporary texts in JCEFP). In the following subsections, we start by presenting the results of the statistical analyses (Section 3.1) before turning to the results from classification (Section 3.2).

### 3.1. Statistical Analysis of Features

For the category of earlier texts we used the data published in Mohseni et al. [7], where the texts of the JEFP corpus were analysed. For contemporary texts we created a series of seven observables for each text in the JCEFP corpus, following the procedure of Mohseni et al. [7]. We determined sentence lengths and the number of specific POS tags in windows of 25 tokens for six POS tags (see Section 2.3). For each series we computed ApEn and ShEn values (Section 2.4). We then compared the text categories using their median values because a Kolmogorov–Smirnov test indicated that some features were not normally distributed. For our statistical comparison we used the non-parametric Mann–Whitney U test.

Table 3 and Table 4 (left-hand side) compare the contemporary bestselling and non-bestselling texts in terms of ApEn and ShEn, respectively. The values of the features for earlier canonical and non-canonical texts are shown on the right-hand side. These data have been taken from Mohseni et al. [7]. To facilitate the comparison of values for each text category/feature combination, the (significantly) higher value of each pair is shown in boldface. For Noun, Verb, Adjective and Preposition, the category of bestseller has higher values than the non-bestselling texts in the contemporary corpus. In both categories, the values for sentence length are not significantly different from each other. Only in one major POS category, i.e., Pronoun, are the values for ApEn and ShEn higher for contemporary non-bestselling texts than for the bestsellers.

If we compare earlier and contemporary texts in the fictional categories, we observe both differences and similarities. In earlier texts the values for all POS tags are higher for canonical texts than for non-canonical texts. Contemporary texts do not show any difference for the category of Adverb. For Pronoun, the value is higher for the non-bestselling texts. In summary, we observe a similar pattern for prepositions and the three POS tags representing major classes of content words, i.e., Noun, Verb and Adjective. Thus, the biggest difference in the comparison of preferred vs. non-preferred texts in the earlier and contemporary periods lies in the distribution of pronouns. Notably, ApEn and ShEn exhibit similar patterns of differences for all comparisons.

Examples of texts with a high degree of unpredictability in the JEFP corpus are *Ulysses* by James Joyce, *The Golden Bowl* by Henry James and *Sartor Resartus* by Thomas Carlyle, showing the highest ApEn values in the category of earlier canonical texts for Noun, Verb and Adjective, respectively. In the bestsellers category among the contemporary texts, *Port Mortuary* by Patricia Cornwell has the highest ApEn value for Noun and the highest ShEn value for Verb. Another prominent example is *Freedom* by Jonathan Franzen, which is the bestseller with the highest ApEn value for Adjective in the corpus.

Both corpora (JEFP and JCEFP) contained non-fictional texts as well. In the Supplementary Materials, Tables S2 and S3 show the results for fictional and non-fictional texts for ApEn and ShEn, respectively. We refer the interested reader to these two supplementary tables, to gain an impression of the comparison between fictional and non-fictional texts. Summarizing the results, there is no uniform pattern in the degree of (un)predictability in fictional or non-fictional texts. For some text properties, such as Verb and Adjective, the values of ApEn and ShEn are higher for fictional than non-fictional texts, while for other text properties, such as Adverb and Pronoun, the opposite pattern can be observed. Moreover, the values of ApEn and ShEn do not correspond to each other in measuring the degree of (un)predictability in the fictional or non-fictional text categories.

Figure S3 in the Supplementary Materials shows a correlation plot for ApEn and ShEn values for all earlier and contemporary text categories, for all text properties. For some text properties, such as Adjective and Adverb, the correlation coefficients are very high, while for others, such as Noun and Verb, they are lower. This finding is related to the difference between the discrimination power of ApEn and ShEn, which becomes visible when we look at the classification results in the next section.

### 3.2. Classification

We extend our analysis of preferred vs. non-preferred texts with a classification tasks. Classification determines the performance of each property/feature in distinguishing the text categories under analysis. For each setting we trained a support vector machine (SVM) with a radial basis function (RBF) kernel. To report the performance of the classification models, we used balanced accuracy, which eliminates the undesired effect of different class sizes in the input data. In the comparison of the classification results we rely on the 5×2CV paired *t*-test [44] with a significance level of α=0.05.

Table 5 shows the balanced accuracy scores for bestselling vs. non-bestselling contemporary texts, for each text property/feature combination. To compare contemporary and earlier texts, we also include the classification results of canonical vs. non-canonical earlier texts, which were published in Mohseni et al. [7] (right-hand side of Table 5).

In the task of classifying bestselling vs. non-bestselling contemporary texts, both ApEn and ShEn perform comparably well, except for Noun and Verb, where ApEn provides a significantly higher accuracy compared to ShEn. Comparing accuracy scores for the two time periods, we observe a shift in the performance of individual text properties, while ApEn of all text properties except Adverb distinguishes canonical from non-canonical earlier texts better than ShEn, the ApEn values of only two text properties in the contemporary texts, i.e., Noun and Verb, provide a better performance compared to ShEn. For other text properties, no significant difference was observed.

The last row of Table 5 shows the performance of classification using all features. No significant difference between the discriminative power of ApEn and ShEn for the bestselling/non-bestselling contemporary texts distinction can be observed. Moreover, the results show that classification using the ApEn values of all text properties cannot distinguish the text categories under study better than ApEn of Noun alone. The difference between the two values is not statistically significant. Using ShEn of all text properties surpasses the performance of all individual ShEn features.

Concerning the results based on all features for earlier texts, ApEn outperforms ShEn with a high margin in the classification of canonical versus non-canonical texts. Taking all text properties into account, the difference between the performance of ApEn and ShEn in the separation of preferred and non-preferred contemporary texts disappears. Nevertheless, the classification accuracy for both features remains comparably high (79.4 and 77.6%, respectively), which confirms that (un)predictability analysis is a promising approach for analysing texts of different aesthetic categories.

## 4. Discussion and Conclusions

Confirming the results obtained by Mohseni et al. [7] for texts from the 19th and early 20th centuries, our study shows that the degree of preference associated with a contemporary text also has correlates in global statistical properties of the text. Generally speaking, preferred texts (bestsellers) are characterized by lower degrees of predictability for most features, as reflected in higher values for the two entropy measures, Shannon Entropy and Approximate Entropy (Table 3 and Table 4).

However, we also found differences between contemporary and earlier texts. The earlier texts were better distinguished by Approximate Entropy than by Shannon Entropy (Table 5) [7]. This shows that the two text categories not only differ in terms of the unpredictability of the part-of-speech rates across windows of text (Shannon Entropy); the part-of-speech rates are also less predictable along the sequential organization of a text (Approximate Entropy). After reading a window of 25 words, a reader has less information about the part-of-speech distribution in the next window of 25 words, in preferred (canonical) texts compared to non-preferred (non-canonical) texts. This is different for the contemporary texts. Approximate Entropy does not globally provide better classification results than Shannon Entropy for this part of the corpus. Only two part-of-speech categories—Noun and Verb—exhibit higher classification accuracy values on the basis of Approximate Entropy than they do based on Shannon Entropy. When all parts of speech as well as sentence length are taken into consideration, there is no significant difference between the classification results (see Table 5). This shows that bestsellers generally exhibit a higher degree of irregularity in the distribution of the linguistic features used for this study than non-bestsellers. The degree of irregularity is not modulated locally, however, and does not depend on the sequential arrangement of structural features.

A second difference between the two time periods is that in the earlier works from the 19th and early 20th centuries, all part-of-speech tags were distributed more unpredictably in the canonical texts than in the non-canonical ones (Table 3 and Table 4). For canonical texts, a low degree of predictability seems to be a general design principle. For contemporary texts, one part of speech, Pronoun, had higher entropy values for the non-bestselling texts compared to the bestselling texts. Moreover, there was no significant difference in the distribution of Adverbs. It seems that only the major classes of content words, Nouns, Verbs and Adjectives as well as Prepositions, whose occurrence correlates with that of nouns, are distributed more unpredictably in bestselling texts as opposed to non-bestselling contemporary texts.

There are at least four possible explanations for the observed differences. The first explanation is based on changes in writing styles. It is well known that narrative styles have changed considerably since the 17th century [25]. This concerns, among other things, the narrator’s visibility and reliability, and the relationship between the narrator and the reader. Moreover, the inventory of registers used in novels has been broadened. For example, the technique of interior monologuing was introduced in modernism [45]. The high degree of unpredictability of POS tags in modern bestsellers, in comparison to non-bestsellers, points to a higher degree of heterogeneity of discourse modes in the former group of texts (Table 3 and Table 4). However, then, the fact that Approximate Entropy does not separate the classes better than Shannon Entropy for all POS tags does seem to show that the sequential arrangement of discourse modes is no less predictable in bestsellers (Table 5). Simplifying this hypothesis, we speculate that bestselling authors draw on a more varied inventory of discourse modes than other authors, but the texts do not exhibit a higher degree of unpredictability as far as the sequential arrangement of these modes is concerned. This hypothesis would require closer inspection of the data, and additional methods that allow us to trace the trajectory of discourse modes across a text.

Related to this first explanation is a second one, which concerns the question of register and genre. Writing styles have not only changed ‘locally’ [25], but there are also shifts in the frequency of literary genres. Among the contemporary texts, specific genres seem to be particularly successful that are rare in the category of canonical texts (e.g., crime stories). As we have no reliable genre classification for our sample, we cannot test for the effect of genre directly. We did, however, conduct an experiment on another corpus, a large collection of fictional texts from several genres (see the Supplementary Materials, Section S3). The results show that distributions of Approximate Entropy and Shannon Entropy vary significantly between genres. However, there is no general pattern across textual properties: there is no genre that exhibits particularly high or low values for all part-of-speech frequencies and sentence length values, while the effect of genre and register as determinants of preference needs to be taken into account without doubt, the results of our preliminary study suggest that they may have a modulating, rather than a direct effect. Further studies are needed to test this assumption.

A third possible explanation for the observed differences between contemporary and earlier texts is provided by the factor of ‘technology’. The process of writing has changed considerably between the earlier period—the 19th and early 20th centuries—and today, while the earlier texts were written either by hand or with a typewriter, contemporary writers can use computers. Texts can easily be edited, and re-edited, and the process of writing requires less planning than it used to. As a consequence, the difference between preferred and non-preferred texts may have decreased, as far as sequential organization is concerned, as the skills of a writer (as the architect of a story) may be less visible in contemporary texts. The general distributions of discourse modes, however, would not be affected by the process of writing, as they seem to be primarily a function of the author’s creativity.

Finally, it is of course conceivable that the two types of preference that we considered—canonization for the earlier texts, sales figures for the contemporary texts—are driven by different forces. The process of canonization is, to a large extent (though not exclusively), driven by academics. It is based on thorough analyses conducted by a community of researchers over an extended period of time. Bestselling books, in contrast, have not gone through this type of filter. For a text to succeed on the book market, it has to be advertised broadly and supported by the media, e.g., with reviews and public discussion. Even though literary critics play an important role in this process, they may have a comparatively small impact on the success of a book (sometimes, negative reviews increase the sales figures, as they lead to controversial public discussion).

From the perspective of empirical aesthetics, it seems conceivable that the design principles of canonical literature—variation both in global distribution and sequential organization—play a less important role in the commercial success of a (contemporary) work. While canonical literature typically targets ‘educated readers’, contemporary bestsellers have a broader target audience—in fact, they tend to target an audience as broad as possible. Aesthetic pleasure varies from reader to reader (see, for example, [46], and for poetry [47,48]). More experienced (or even professional) readers may take pleasure in reading less predictable texts than less experienced readers do.

Unfortunately, we cannot use the same type of operationalization for preference for contemporary and earlier texts, as sales figures (at the time of publication) are not available for the canonical texts (and today’s sales figures are, again, influenced by canonization), and because contemporary texts are too young to be canonized. An alternative way of measuring preference for contemporary texts may be literary prizes. As mentioned in the Section 1, the Nobel Prize has been used as an indicator of preference [4,6]. A comparison between our data and Nobel prize winning books is another project that would broaden our understanding of structural reflexes of preference, and of preference itself.

The program of computational textual aesthetics has been heavily influenced by relevant studies from other domains. For example, statistical properties of (time) series have been analysed for music [31], poetry [49], and even bird song [50]. Measures such as autocorrelation, variability, surprise and predictability have also been used to predict musical preferences in humans [30,51]. As our own work has been influenced by the work on vision, we conclude with a remark on how our results relate to the visual domain. Here, basic perceptual features are also richer and more variable (or less predictable) in artworks than in many types of non-art images. Examples include the spatial distribution of luminance and colour edges across an image [52] and other basic visual features, such as edge orientation, spatial frequency tuning and colour–opponent spatial organization [12,53]. Whether a high degree of variation in such basic perceptual features is universal across aesthetic domains (visual art, literature, dance, music, etc.) is unclear at present.

In relevant studies, perceptual (structural) differences between traditional visual artworks and contemporary art have been observed. With the rise of modern art at the end of the 19th century, the pattern of image properties in visual artworks diversified [54,55]. In parallel, perceptual features that mediate the sensual beauty of artworks became less central for aesthetic judgements. Instead, image content and cultural context emerged as guides of what beholders prefer [56].

We speculate that there are parallels between aesthetic experience in the visual domain and in reading. In both domains, aesthetic preference seems to be related to the interplay between predictability and surprise. Our results are compatible with the hypothesis that the determinants of aesthetic experience in reading, like those in vision, are partly time-invariant, and partly culturally determined. A certain amount of variability and unpredictability, reflected in Approximate Entropy and Shannon Entropy in the present, study seems to be a good candidate for a time-invariant factor. However, in order to gain a better understanding of the determinants of preference in reading, several follow-up studies as sketched above will be needed.

## Figures and Tables

**Table 1:** Text categories in the Jena Corpus of Expository and Fictional Prose (JEFP), version 2.0. The table shows, for each text category, the number of texts and the mean text length, measured in tokens, ±standard deviation. Data are from the study by Mohseni et al. [7].

Category	Number of Texts	Length ($\times 10^{3}$)
Canonical (preferred)	76	199 ± 96
Non-Canonical (non-preferred)	130	111 ± 56
Non-Fictional	185	171 ± 178

**Table 2:** Text categories in the Jena Corpus of Contemporary Expository and Fictional Prose (JCEFP). The table shows, for each text category, the number of texts and the mean text length, measured in tokens, ±standard deviation.

Category	Number of Texts	Length ($\times 10^{3}$)
Bestseller	93	153 ± 90
Non-Bestseller	110	105 ± 39
Non-Fictional	122	142 ± 84

**Table 3:** Median values of Approximate Entropy (ApEn) for all text properties and for all fictional text categories. ApEn values for contemporary bestselling (*N* = 94) vs. non-bestselling (*N* = 110) texts, and for canonical (*N* = 76) vs. non-canonical (*N* = 130) texts. The asterisks indicate whether the differences between the two text categories in the earlier or contemporary periods are statistically significant (Mann–Whitney U test; ns, not significant; *, p≤0.05; **, p≤0.01; and ***, p≤0.001). Values that are significantly higher within a pair of columns are shown in boldface. The 95% confidence intervals for the median (according to [43]) are shown in parentheses. The data for earlier texts are from the study by Mohseni et al. [7].

	Contemporary		Earlier	
Text Property	Bestseller	Non-Bestseller	Canonical	Non-Canonical
Sentence Length	1.99 (1.95, 2.02)	2.01 (1.99, 2.04) ^ns^	1.86 (1.83, 1.89)	1.87 (1.86, 1.90) ^ns^
Noun	**1.93 (1.921, 1.934)**	1.85 (1.84, 1.86) ***	**1.89 (1.88, 1.91)**	1.83 (1.81, 1.84) ***
Verb	**1.74 (1.730, 1.742)**	1.70 (1.68, 1.71) ***	**1.75 (1.73, 1.76)**	1.70 (1.69, 1.71) ***
Adjective	**1.40 (1.38, 1.41)**	1.36 (1.34, 1.38) **	**1.50 (1.49, 1.52)**	1.45 (1.43, 1.48) ***
Adverb	1.50 (1.47, 1.53)	1.51 (1.50, 1.52) ^ns^	**1.51 (1.49, 1.53)**	1.48 (1.46, 1.49) **
Pronoun	1.71 (1.69, 1.73)	**1.73 (1.71, 1.74)** *	**1.74 (1.71, 1.76)**	1.681 (1.675, 1.691) ***
Preposition	**1.63 (1.62, 1.64)**	1.61 (1.60, 1.62) ***	**1.71 (1.70, 1.72)**	1.67 (1.66, 1.68) ***

**Table 4:** Median values of Shannon Entropy (ShEn) for all text properties and for all fictional text categories. ShEn values for contemporary bestselling (*N* = 94) vs. non-bestselling (*N* = 110) texts, and for canonical (*N* = 76) vs. non-canonical (*N* = 130) texts. The asterisks indicate whether the differences between the two text categories in the earlier or contemporary periods are statistically significant (Mann–Whitney U test; ns, not significant; *, p≤0.05; **, p≤0.01; and ***, p≤0.001). Values that are significantly higher within a pair of columns are shown in boldface. The 95% confidence intervals for the median (according to [43]) are shown in parentheses. The data for earlier texts are from the study by Mohseni et al. [7].

	Contemporary		Earlier	
Text Property	Bestseller	Non-Bestseller	Canonical	Non-Canonical
Sentence Length	3.42 (3.39, 3.46)	3.36 (3.31, 3.39) ^ns^	3.96 (3.88, 4.05)	3.96 (3.87, 4.08) ^ns^
Noun	**2.09 (2.08, 2.11)**	1.99 (1.77, 2.02) ***	**2.00 (1.99, 2.02)**	1.97 (1.95, 1.98) ***
Verb	**1.80 (1.78, 1.81)**	1.77 (1.767, 1.789) ***	**1.80 (1.79, 1.81)**	1.777 (1.772, 1.783) ***
Adjective	**1.43 (1.41, 1.45)**	1.39 (1.37, 1.42) **	**1.54 (1.53, 1.55)**	1.49 (1.47, 1.53) ***
Adverb	1.53 (1.49, 1.56)	1.54 (1.53, 1.57) ^ns^	**1.54 (1.51, 1.55)**	1.51 (1.49, 1.53) *
Pronoun	1.80 (1.79, 1.81)	**1.82 (1.81, 1.84)** ***	**1.83 (1.80, 1.84)**	1.78 (1.77, 1.79) ***
Preposition	**1.67 (1.66, 1.68)**	1.66 (1.64, 1.67) *	**1.75 (1.74, 1.77)**	1.73 (1.72, 1.74) ***

**Table 5:** Balanced accuracy of classification (%) for the single features for the bestselling/non-bestselling contemporary texts distinction and for the canonical/non-canonical early texts distinction. Values that are significantly higher within a pair of columns are shown in boldface. Wherever the results are not significantly better than random accuracy (50%), we mark the result with a dagger ^†^. The data for earlier texts are from the study by Mohseni et al. [7].

	Bestselling vs. Non-Bestselling		Canonical vs. Non-Canonical	
	ApEn	ShEn	ApEn	ShEn
Sentence Length	53.6 ± 3.1	53.8 ± 3.0	**54.0 ± 1.6**	50.0 ± 1.0 ^†^
Noun	**80.4 ± 3.4**	72.9 ± 2.7	**73.6 ± 2.9**	60.0 ± 4.5
Verb	**67.7 ± 3.7**	62.7 ± 2.5	**71.3 ± 3.4**	56.2 ± 3.8
Adjective	56.2 ± 3.2	57.4 ± 3.3	**55.2 ± 2.5**	51.5 ± 2.7 ^†^
Adverb	53.6 ± 2.2	51.3 ± 2.6 ^†^	51.6 ± 1.4 ^†^	51.0 ± 1.5 ^†^
Pronoun	57.6 ± 1.8	58.1 ± 1.9	**68.0 ± 1.7**	63.8 ± 1.8
Preposition	57.8 ± 2.6	53.5 ± 2.2	**69.1 ± 2.4**	59.7 ± 1.7
All	79.4 ± 4.2	77.6 ± 2.4	**77.3 ± 2.6**	68.5 ± 2.3

## Data Availability

The data presented in this study are available on request from the corresponding author. The data are not publicly available due to copyright restrictions.

## Supplementary Materials

The following are available online at https://www.mdpi.com/article/10.3390/e25030486/s1, Section S1: Previous Work on the Temporal Analysis of Language; Section S2: Approximate Entropy; Section S3: Effects of Genre; Figure S1. Boxplot of ApEn for all genres and for all text properties; Figure S2. Boxplot of ShEn for all genres and for all text properties; Table S1: List of texts in the Jena Corpus of Contemporary Expository and Fictional Prose (JCEFP Corpus); Figure S3: Approximate Entropy vs. Shannon Entropy for each text category and for each text property; Table S2: Median values of Approximate Entropy (ApEn) for all text properties and for all fictional text categories; Table S3: Median values of Shannon Entropy (ShEn) for all text properties and for all fictional text categories. Refs. [7,43,57,58,59,60,61,62,63,64,65,66,67,68,69,70,71,72,73] are cited in the Supplementary Materials.
