# Supplementary material for: Comparative Analysis of Preference in Contemporary and Earlier Texts Using Entropy Measures
Source: Entropy (Basel). 2023 Mar 10;25(3):486. doi: 10.3390/e25030486 (PMC10048171; doi:10.3390/e25030486)
Supplement: Supplementary file 1 [file entropy-25-00486-s001.zip › entropy-2234510-supplementary.pdf]

Supplementary Materials: Comparative Analysis of Preference in Contemporary and Earlier Texts Using Entropy Measures

1. Previous Work on Temporal Analysis of Language

Kumar *et al.* [1] applied the Kullback-Leibler divergence to histograms derived from the language model of documents to determine the time period of short stories published between 1798 and 2008. Their approach predicted dates of publication, though within a wide range of temporal difference. Garcia-Fernandez *et al.* [2] used several external resources, e.g. Wikipedia, to detect the publication date of article excerpts from seven French newspapers published from the early 19th century to the middle of the 20th century. Their system correctly detected the year of publication for up to 14% of documents, and the decade of publication for up to 42% of the documents. Ciobanu *et al.* [3] applied temporal text classification to Romanian novels, showing that a high classification accuracy can be achieved with bag-of-word features.

Štajner and Zampieri [4] proposed a supervised method to study stylistic changes in Portuguese historical texts spanning a range from the 17th century to the early 20th century. They revealed a noticeable shift in lexical diversity and lexical richness of texts written in the 19th and 20th centuries, compared to the texts from the two preceding centuries. Gómez-Adorno *et al.* [5] analyzed changes in the writing styles of seven authors using stylometric features. They achieved a high level of accuracy in the detection of writing stages for works by some authors. Efremova *et al.* [6] worked on Dutch historical notary acts spanning a period of more than six centuries. First, they identified time periods based on historical events. Then, using Term Frequency–Inverse Document Frequency (TF-IDF) features and spectral clustering, they classified texts into these periods, reaching a high accuracy level.

Liebeskind and Liebeskind [7] applied neural classifiers to historical Hebrew texts from four different periods. They showed that neural networks outperformed classic machine learning algorithms in the task of period classification. Gopidi and Alam [8] studied stylistic differences between prose and poem in two different time spans of 1870–1920 and 1970–2019. They combined quantitative analyses with interpretation based on close reading. Using features derived from grammatical properties, meter and rhyme, they concluded on the basis of their classification results that modern poetry is more similar to prose, in comparison to older poetry and prose. Lagutina *et al.* [9] studied rhythm in 300 English and Russian prosaic texts dating from the 19th century to the 21st century. They found that rhythm figures change with time and can be regarded as a determinant of an author's style. This approach was extended in Lagutina and Manakhova [10], who analyzed not only rhythm features but also low-level features, i.e. at the level of the word and symbol. They compared stylometric features of texts from each decade and revealed that rhythm features have changed more than other features in the texts under analysis. They also found that the average lengths of sentences decreased in Russian texts in a wave form during the last two centuries, while the average word length increased consistently. Degaetano-Ortlieb [11] used lexical and grammatical models to explore stylistic variation of different groups of language users. She showed that temporal stylistic changes across genders and classes of society can be captured using Relative Entropy (Kullback-Leibler Divergence).

Diachronic analysis of scientific texts have also been addressed in previous studies. For example, Fankhauser *et al.* [12] used topic modeling to monitor topic developments in a corpus of the Royal Society of London. Unsurprisingly, their observations showed that scientific topics have diversified over time, while individual documents have been more specialized in terms of topics. Bizzoni *et al.* [13] proposed to investigate diachronic language changes using Relative Entropy as a measure of diversification. They analyzed scientific English texts published in a period longer than 250 years and showed evidence of register formation and, at the same time, diversification in word usage. Wang *et al.* [14] explored temporal variation of linguistic structures by applying Kolmogorov complexity to different types of scientific texts. Their analysis showed that while the scientific lexicon has been enriched during the time period analyzed, the language complexity has declined in favor of grammatical simplification. There are also studies which focused on more specific language structures in scientific writing, e.g. temporal expressions [15] and *wh*-words [16].

2. Approximate Entropy

For series $X = x(1), \cdots, x(n)$, sub-sequences of length m, $y_i^m = [x(i), \cdots, x(i + (m-1))]$, and tolerance r, Approximate Entropy (ApEn) is computed as follows:

1. Compute Chebyshev distance between each sub-sequence y_i^m and y_j^m:

$$d_{i,j}^m = \max_k |y_i^m(k) - y_j^m(k)|$$

2. Using the Heaviside function, $\mathbb{1}(.)$, whose value is zero for negative arguments and one for positive arguments, for each sub-sequence y_i^m, compute:
$$C_i^m(r) = \frac{1}{n-m+1} \sum_{j=1}^{n-m+1} \mathbb{1}(r - d_{i,j}^m)$$
3. Compute
$$\phi^m(r) = \frac{1}{n-m+1} \sum_{i=1}^{n-m+1} \log(C_i^m(r))$$
4. Repeat step 1 to 3 for sub-sequences of length $m+1$ to compute $\phi^{m+1}(r)$.
5. Calculate ApEn as
$$\texttt{ApEn}(m,r) = \phi^m(r) - \phi^{m+1}(r)$$

3. Effects of Genre

Genre characteristics may have some effects on structural properties of texts. We analyzed Approximate Entropy (ApEn) and Shannon Entropy (ShEn) of series of 6 POS-tags (Noun, Verb, Adjective, Adverb, Pronoun, Preposition) and sentence length in various genres. We used texts from the US Novel corpus [17]. We analyzed texts written from 1980 to 2000, which are more similar to the contemporary texts in our corpus (JCEFP) in terms of publication time. We did not include multi-labeled texts and we analyzed categories which contained at least 50 texts. As a result, 3168 texts from 7 categories were selected. Figures S1 and S2 show the results for ApEn and ShEn, respectively. As the plots demonstrate, the distribution of ApEn and ShEn are different for the text categories. An ANOVA test confirmed that the distribution of ApEn and ShEn differed between the genres for all text properties. However, the ranking of the genres changes for different text properties and none of them consistently shows the highest or lowest values compared to the other genres.

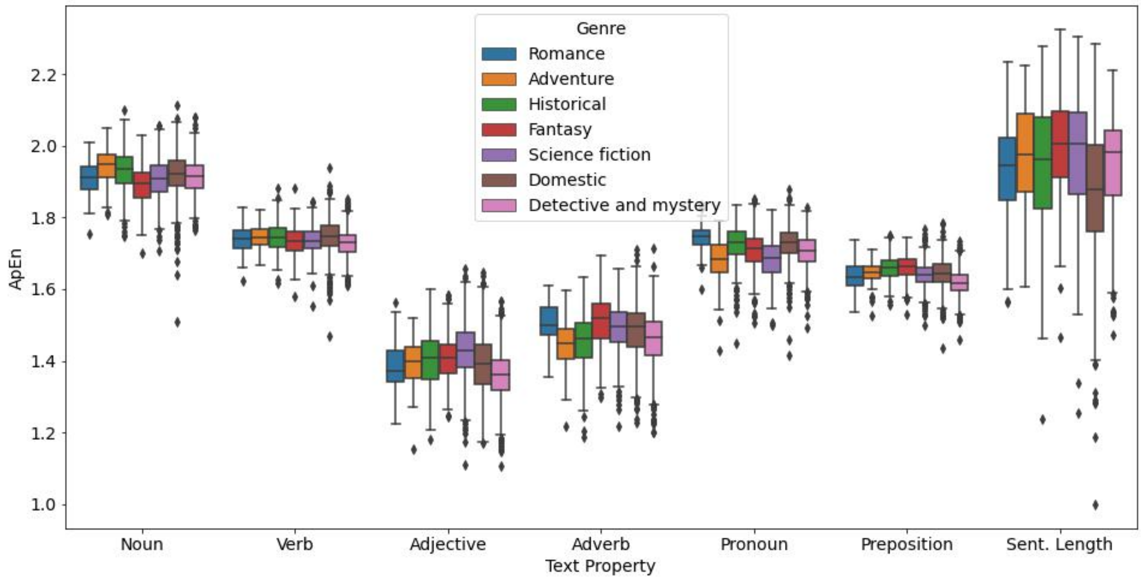

Figure S1. Boxplot of ApEn for all genres and for all text properties.

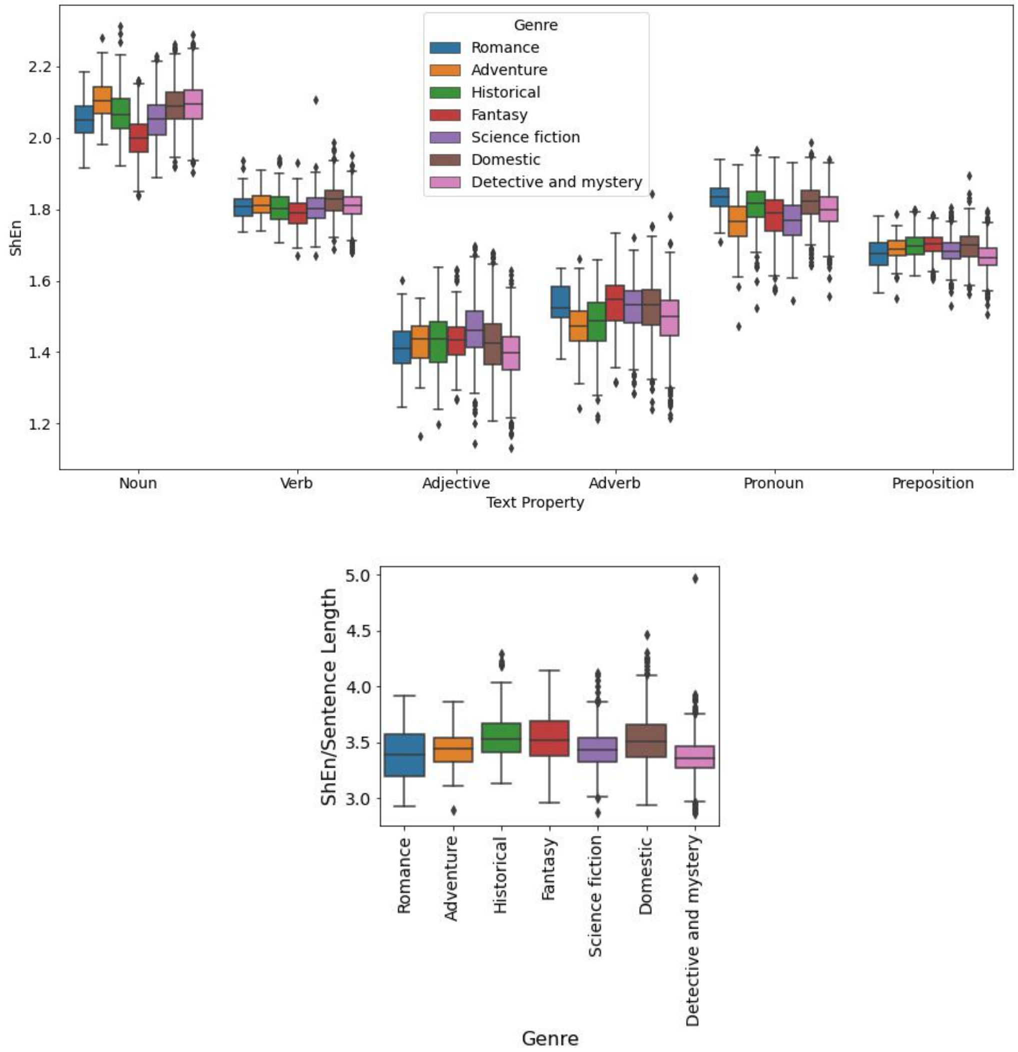

Figure S2. Boxplot of ShEn for all genres and for all text properties. The plot of sentence length is separated as its values are in a different range.

4. Tables and Figures

Table S1: List of texts in the Jena Corpus of Contemporary Expository and Fictional Prose (JCEFP Corpus). Bestseller texts were selected from lists of the New York Times Best Sellers. Corpus of non-bestseller texts were downloaded from www.smashwords.com. Non-fictional texts were selected from various sources.

	**Title**	**Author(s)**	**Year of Publication**	**Category**
1	11.22.63	Stephen King	2011	Bestseller
2	5th Horseman	James Patterson, Maxine Paetro	2006	Bestseller
3	A Dance with Dragons	George R. R. Martin	2011	Bestseller
4	A Man Called Ove	Fredrik Backman	2012	Bestseller
5	A Thousand Splendid Suns	Khaled Hosseini	2007	Bestseller
6	Alex Cross	James Patterson	2009	Bestseller
7	All the Light We Cannot See	Anthony Doerr	2014	Bestseller
8	Allegiant	Veronica Roth	2013	Bestseller
9	Angels and Demons	Dan Brown	2000	Bestseller
10	At First Sight	Nicholas Sparks	2005	Bestseller
11	Book of the Dead	Patricia Cornwell	2008	Bestseller
12	Catching Fire	Suzanne Collins	2009	Bestseller
13	Cell	Stephen King	2006	Bestseller

Continued on next page

Table S1 – *Continued from previous page*

	Title	Author(s)	Year of Publication	Category
14	Cross	James Patterson	2006	Bestseller
15	Cross Country	James Patterson	2008	Bestseller
16	Cross Fire	James Patterson	2011	Bestseller
17	Dead or Alive	Tom Clancy, Grant Blackwood	2010	Bestseller
18	Dead Rechoning	Charlaine Harris	2001	Bestseller
19	Dear John	Nicholas Sparks	2006	Bestseller
20	Desecration	Tim F. LaHaye, Jerry B. Jenkins	2001	Bestseller
21	Doctor Sleep	Stephen King	2013	Bestseller
22	Double Cross	James Patterson	2007	Bestseller
23	Dreamcatcher	Stephen King	2001	Bestseller
24	Eleven on Top	Janet Evanovich	2005	Bestseller
25	Everythings Eventual	Stephen King	2002	Bestseller
26	Fearless Fourteen	Janet Evanovich	2008	Bestseller
27	Fifty Shades Darker	EL James	2011	Bestseller
28	Fifty Shades Freed	EL James	2012	Bestseller
29	Fifty Shades of Grey	EL James	2011	Bestseller
30	Fifty Shades of Louisa May	L. M. Anonymous	2012	Bestseller
31	Finger Lickin Fifteen	Janet Evanovich	2009	Bestseller
32	Four Blind Mice	James Patterson	2001	Bestseller
33	Freedom	Jonathan Franzen	2010	Bestseller
34	Full Dark, No Stars	Stephen King	2010	Bestseller
35	Go Set a Watchman	Harper Lee	2015	Bestseller
36	Hannibal Rising	Thomas Harris	2006	Bestseller
37	Inferno	Dan Brown	2013	Bestseller
38	Insurgent	Veronica Roth	2012	Bestseller
39	Jack Ryan12 The Teeth Of The Tiger	Tom Clancy	2003	Bestseller
40	Kill Alex Cross	James Patterson	2011	Bestseller
41	Light from Heaven	Jan Karon	2005	Bestseller
42	Liseys Story	Stephen King	2006	Bestseller
43	Locked On	Tom Clancy, Mark Greaney	2011	Bestseller
44	London Bridges	James Patterson	2004	Bestseller
45	Mary Mary	James Patterson	2004	Bestseller
46	Micro	Michael Crichton, Richard Preston	2011	Bestseller
47	Mockingjay	Suzanne Collins	2010	Bestseller
48	Next	Michael Crichton	2006	Bestseller
49	Pirate Latitudes	Michael Crichton	2009	Bestseller
50	Port Mortuary	Patricia Cornwell	2010	Bestseller
51	Prey	Michael Crichton	2002	Bestseller
52	Red Rabbit	Tom Clancy	2002	Bestseller
53	Safe Haven	Nicholas Sparks	2010	Bestseller
54	Sizzling Sixteen	Janet Evanovich	2010	Bestseller
55	Skipping Christmas	John Grisham	2001	Bestseller
56	Smokin Seventeen	Janet Evanovich	2011	Bestseller
57	State of Fear	Michael Crichton	2004	Bestseller
58	The 6th Target	James Patterson, Maxine Paetro	2007	Bestseller
59	The Appeal	John Grisham	2008	Bestseller
60	The Associate	John Grisham	2009	Bestseller
61	The Best of Me	Nicholas Sparks	2010	Bestseller
62	The Big Bad Wolf	James Patterson	2003	Bestseller
63	The Broker	John Grisham	2005	Bestseller
64	The Choice	Nicholas Sparks	2007	Bestseller
65	The Christmas Sweater	Glenn Beck	2008	Bestseller
66	The Confession	John Grisham	2010	Bestseller
67	The Da Vinci Code	Dan Brown	2003	Bestseller
68	The Darkest Evening of the Year	Dean Koontz	2007	Bestseller
69	The Fault in Our Stars	John Green	2012	Bestseller
70	The Five People You Meet in Heaven	Mitch Albom	2003	Bestseller
71	The Girl on the Train	Paula Hawkins	2015	Bestseller
72	The Girl Who Kicked the Hornets Nest	Stieg Larsson	2007	Bestseller
73	The Help	Kathryn Stockett	2009	Bestseller
74	The Historian	Elizabeth Kostova	2005	Bestseller
75	The Host	Stephenie Meyer	2008	Bestseller
76	The House of Hades	Rick Riordan	2013	Bestseller

Continued on next page

Table S1 – *Continued from previous page*

	Title	Author(s)	Year of Publication	Category
77	The Hunger Games	Suzanne Collins	2010	Bestseller
78	The King of Torts	John Grisham	2003	Bestseller
79	The Last Song	Nicholas Sparks	2009	Bestseller
80	The Litigators	John Grisham	2011	Bestseller
81	The Lost Symbol	Dan Brown	2009	Bestseller
82	The Lovely Bones	Alice Sebold	2002	Bestseller
83	The Mark of Athena	Rick Riordan	2012	Bestseller
84	The Martian	Andy Weir	2014	Bestseller
85	The Quickie	James Patterson, Michael Ledwidge	2009	Bestseller
86	The Remnant	Tim LaHaye, Jerry B. Jenkins	2002	Bestseller
87	The Rule of Four	Ian Caldwell and Dustin Thomason	2004	Bestseller
88	The Shelters of Stone	Jean M. Auel	2002	Bestseller
89	The Story of Edgar Sawtelle	David Wroblewski	2008	Bestseller
90	The Wedding	Nicholas Sparks	2003	Bestseller
91	True Believer	Nicholas Sparks	2005	Bestseller
92	Twelve Sharp	Janet Evanovich	2006	Bestseller
93	Your Heart Belongs to Me	Dean Ray Koontz	2008	Bestseller
94	A Highland Affair	Richard F. Jones	2018	Non-Bestseller
95	A Long, Cool Rain	Linda Seed	2017	Non-Bestseller
96	A Unicorn's Memoir	Stephanie Menges	2020	Non-Bestseller
97	After the Fire	Kathryn Shay	2003	Non-Bestseller
98	After the Fog: A Novel	Kathleen Shoop	2012	Non-Bestseller
99	An English Visitor	Graham Wilson	2007	Non-Bestseller
100	An Ignorant Witch	E M Graham	2019	Non-Bestseller
101	Ash and Water	Everleigh Miles	2020	Non-Bestseller
102	Awakened	Brenda K. Davies	2012	Non-Bestseller
103	Bad Choices Make Good Stories: Going to New York	Oliver Markus Malloy	2017	Non-Bestseller
104	Beautiful Secret	Claire Raye	2019	Non-Bestseller
105	Beg For You: A Small Town Romance	Sherilee Gray	2019	Non-Bestseller
106	Breaking the Rules	Ruth Ann Nordin	2020	Non-Bestseller
107	Bridge Through Time	Scott Spotson	2014	Non-Bestseller
108	Cactus Island	William Manchee	2006	Non-Bestseller
109	Case of the One-Eyed Tiger	Jeffrey M. Poole	2020	Non-Bestseller
110	Christmas Magic	Alexandra Moody	2018	Non-Bestseller
111	Clocks Locks and Danger	Lizzie Lewis	2020	Non-Bestseller
112	Co-Ed	Rachel Van Dyken	2018	Non-Bestseller
113	Cole	Tory Richards	2019	Non-Bestseller
114	Crocodile Man	Graham Wilson	2017	Non-Bestseller
115	Darkhouse	Karina Halle	2011	Non-Bestseller
116	Delusions	Christina Smith	2012	Non-Bestseller
117	Dragma's Keep	Vance Pumphrey	2015	Non-Bestseller
118	Dreaming of You	S.E. Felida	2020	Non-Bestseller
119	Duly Noted	H.M. Shander	2016	Non-Bestseller
120	Dying for a Living	Kory M. Shrum	2014	Non-Bestseller
121	Elfin	Quinn Loftis	2012	Non-Bestseller
122	Eternally Bound	Brenda K. Davies	2016	Non-Bestseller
123	Ever Onward	Wayne Mee	2011	Non-Bestseller
124	Everything we Lost	Kate Smith	2016	Non-Bestseller
125	Falling For You	Leeanna Morgan	2018	Non-Bestseller
126	Falling Into The Black	Lauren Runow	2017	Non-Bestseller
127	Fated Dreams	Christina Smith	2012	Non-Bestseller
128	Fighting Destiny	Amelia Hutchins	2013	Non-Bestseller
129	Fire Song	Val St. Crowe	2016	Non-Bestseller
130	Frey	Melissa Wright	2019	Non-Bestseller
131	Genesis Code	Eliza Green	2012	Non-Bestseller
132	Girl in a Cage	Graham Wilson	2019	Non-Bestseller
133	Governor	Lesli Richardson	2018	Non-Bestseller
134	Hellfire - Treachery	Simon Goodson	2020	Non-Bestseller
135	Human Intelligence	Klaus Marre	2013	Non-Bestseller
136	I Woke Up Feeling Thailand	D. Bruno Starrs	2012	Non-Bestseller
137	Ice Homme	Vance Pumphrey	2015	Non-Bestseller

Continued on next page

Table S1 – *Continued from previous page*

	Title	Author(s)	Year of Publication	Category
138	In Defense of Mankind	Ron L. Carter, H.R. Carter	2019	Non-Bestseller
139	Just One Kiss	Jami Rogers	2020	Non-Bestseller
140	Just Visiting	Graham Wilson	2015	Non-Bestseller
141	Killing Me Softly	Bianca Sloane	2012	Non-Bestseller
142	King's Crown	Marie Johnston	2020	Non-Bestseller
143	Last Breath	Greg Tuck	2015	Non-Bestseller
144	Legacy of Darkness: Undercover Mistress	Dai Fuse	2020	Non-Bestseller
145	Like a Memory	Abbi Glines	2017	Non-Bestseller
146	Little Lost Girl	Graham Wilson	2011	Non-Bestseller
147	Lost	Jodi Kae	2016	Non-Bestseller
148	Lost Girl	Chanda Hahn	2016	Non-Bestseller
149	Lost in Me	Lexi Ryan	2014	Non-Bestseller
150	Loveoid	J.L. Morin	2020	Non-Bestseller
151	Moonstone	Linda Seed	2015	Non-Bestseller
152	Mystic Mayhem	Sally J. Smith	2015	Non-Bestseller
153	No More Tears	Sandy Appleyard	2020	Non-Bestseller
154	Nowhere Man	Graham Wilson	2020	Non-Bestseller
155	Of Beast and Beauty	Chanda Hahn	2019	Non-Bestseller
156	Our Broken Pieces	M.E. Clayton	2020	Non-Bestseller
157	Pierced	Sydney Landon	2015	Non-Bestseller
158	Possession	Graham Wilson	2018	Non-Bestseller
159	Prince of Wolves	Quinn Loftis	2013	Non-Bestseller
160	Ragnarok Conspiracy	Rob J. Meijer	2018	Non-Bestseller
161	Red Hot Mama	Reagan McDaniels	2021	Non-Bestseller
162	Redemption Lake	Susan Clayton-Goldner	2017	Non-Bestseller
163	Return of the Breaker	Graham Wilson	2018	Non-Bestseller
164	Riley 's Secret	Christina Smith	2012	Non-Bestseller
165	Riley 's Torment	Christina Smith	2013	Non-Bestseller
166	Rise of the Gladiator	Cheree Alsop	2020	Non-Bestseller
167	Rosebloom	Christine Keleny	2008	Non-Bestseller
168	Safe Haven	Leeanna Morgan	2016	Non-Bestseller
169	Saving Grace	Sandy James	2013	Non-Bestseller
170	Sealed with a Kiss	Leeanna Morgan	2016	Non-Bestseller
171	Seeking Dr. Magic	Scott Spotson	2018	Non-Bestseller
172	Shadow Phantoms	H.P. Mallory	2020	Non-Bestseller
173	Silent Star	James F. David	2014	Non-Bestseller
174	Some Call it Love	Sarah Peis	2018	Non-Bestseller
175	Soul of the Dragon	Natalie J. Damschroder	2012	Non-Bestseller
176	The American Terrorist	Ron L. Carter	2012	Non-Bestseller
177	The Broken	Igor Ljubuncic	2013	Non-Bestseller
178	The Bulldog	Tricia Andersen	2019	Non-Bestseller
179	The Diary	Graham Wilson	2014	Non-Bestseller
180	The Dragon Question	L. Darby Gibbs	2018	Non-Bestseller
181	The Dragon's Slave	Lacey St. Sin	2016	Non-Bestseller
182	The Empty Place	Graham Wilson	2014	Non-Bestseller
183	The Heartbreaker	Tricia Andersen	2015	Non-Bestseller
184	The House on Persimmon Road	Jackie Weger	2014	Non-Bestseller
185	The Library of Antiquity	Vance Pumphrey	2013	Non-Bestseller
186	The Mystery of the Hidden Jewels	Carrie Cross	2014	Non-Bestseller
187	The Old Balmain House	Graham Wilson	2011	Non-Bestseller
188	The Platinum Dragon	Vance Pumphrey	2015	Non-Bestseller
189	The Prophecy	Jeffrey M. Poole	2012	Non-Bestseller
190	The Storm Inside	Alexis Anne	2013	Non-Bestseller
191	The Strange Life of Brandon Chambers	Scott Spotson	2020	Non-Bestseller
192	The Truth About James	Sarah Tork	2014	Non-Bestseller
193	The Valkyrie	L K Walker	2018	Non-Bestseller
194	The Watchers	Lynnie Purcell	2011	Non-Bestseller
195	Theft of the Giant's Soul	Mark Cheverton	2019	Non-Bestseller
196	Trapped	Graham Wilson	2017	Non-Bestseller
197	Trigger	L. P. Dover	2017	Non-Bestseller
198	True Colors	Melissa Pearl	2014	Non-Bestseller
199	Unlucky Charm	Kimberly Gordon	2017	Non-Bestseller
200	Wagon Trail Bride	Ruth Ann Nordin	2016	Non-Bestseller

Continued on next page

Table S1 – *Continued from previous page*

	Title	Author(s)	Year of Publication	Category
201	Wild On You	Justiss Alliance	2014	Non-Bestseller
202	Witch's Bell	Odette C. Bell	2010	Non-Bestseller
203	Yesterday's Sins	Shirley Wine	2012	Non-Bestseller
204	A Compendium of Philosophical Concepts and Methods	Peter S. Fosl: JULIAN BAGGINI	2020	Nonfictional
205	A Grand Origin for Grand Canyon	Michael Oard	2014	Nonfictional
206	Act Natural: A Cultural History of Misadventures in Parenting	Jennifer Traig	2019	Nonfictional
207	Aesthetics Volume II	Dietrich von Hildebrand	2019	Nonfictional
208	AI Ethics	Mark Coeckelbergh	2020	Nonfictional
209	Anatomy 101: From Muscles and Bones to Organs and Systems Your Guide to How the Human Body Works	Kevin Langford	2015	Nonfictional
210	Aristotle's ladder, Darwin's tree : the evolution of visual metaphors for biological order	J. David Archibald	2014	Nonfictional
211	Art and Architecture of Viceregal Latin America 1521-1821	Kelly Donahue Wallace	2008	Nonfictional
212	Awkward: The Science of Why We re Socially Awkward and Why That s Awesome	Ty Tashiro	2017	Nonfictional
213	Balance: A Dizzying Journey Through the Science of Our Most Delicate Sense	Carol Svec	2017	Nonfictional
214	Biochemistry	Denise R. Ferrier PhD	2013	Nonfictional
215	Biostatistics: The Bare Essentials	Geoffrey R. Norman David L. Sreiner	2014	Nonfictional
216	Bird Sense: What It s Like to Be a Bird	Tim Birkhead	2012	Nonfictional
217	Blind Descent: The Quest to Discover the Deepest Place on Earth	James M. Tabor	2010	Nonfictional
218	Cannibalism: A Perfectly Natural History	Bill Schutt	2017	Nonfictional
219	City Shaped Churches: Planting Churches in a Global Era	Linda Bergquist Michael Crane	2018	Nonfictional
220	Climate Action Planning: A Guide to Creating Low Carbon Resilient Communities	Michael R. Boswell, Adrienne I. Greve Tammy L. Seale	2019	Nonfictional
221	Communication and Capitalism: A Critical Theory	Christian Fuchs	2020	Nonfictional
222	Competition Overdose: How Free Market Mythology Transformed Us from Citizen Kings to Market Servants	Maurice E. Stucke: Ariel Ezrachi	2020	Nonfictional
223	Conflict and Contest in Nietzsche s Philosophy	Herman Siemens: James Pearson	2019	Nonfictional
224	Coping with Trauma Related Dissociation: Skills Training for Patients and Therapists	Suzette Boon, Kathy Steele, Onno van der Hart	2011	Nonfictional
225	Copyright Law for Librarians and Educators	Kenneth D. Crews	2011	Nonfictional
226	Cosmic DNA at the Origin: A Hyperdimension before the Big Bang: the Infinite Spiral Staircase Theory	Chris H. Hardy	2015	Nonfictional
227	Dazzled and Deceived: Mimicry and Camouflage	Peter Forbes	2011	Nonfictional
228	Decriminalizing Domestic Violence: A Balanced Policy Approach to Intimate Partner Violence	Leigh Goodmark	2018	Nonfictional
229	Dog Behaviour Evolution and Cognition	Adam Miklosi	2015	Nonfictional
230	Drawn from Paradise: The Natural History Art and Discovery of the Birds of Paradise with Rare Archival Art	David Attenborough, Errol Fuller	2012	Nonfictional
231	EcoCities: Rebuilding Cities in Balance with Nature	Richard Register	2006	Nonfictional
232	Emotional Intelligence: Emotional Mastery Influence	Modern Psychology Publishing	2019	Nonfictional
233	Enforcement of Maritime Claims	David Jackson	2005	Nonfictional
234	Environmental and Low Temperature Geochemistry	Peter Ryan	2019	Nonfictional
235	Epistemology and the Psychology of Human Judgment	Michael A. Bishop, J. D. Trout	2005	Nonfictional
236	Essentials of Environmental Health	Robert H. Friis	2012	Nonfictional
237	Ethnosociology: The Foundations	Alexander Dugin	2019	Nonfictional
238	Five Pillars of the Mind: Redesigning Education to Suit the Brain	Tracey Tokuhama Espinosa	2019	Nonfictional
239	Fundamentals of Structural Mechanics Dynamics and Stability	A.I. Rusakov	2020	Nonfictional
240	Geology by Design: Interpreting Rocks and their Catastrophic Record	Carl R. Froede	2007	Nonfictional
241	Global Sales and Contract Law	Ingeborg Schwenzer, Christopher Kee, Pascal Hachem	2012	Nonfictional

Continued on next page

Table S1 – *Continued from previous page*

	Title	Author(s)	Year of Publication	Category
242	Happy City: Transforming Our Lives Through Urban Design	Charles Montgomery	2015	Nonfictional
243	Hegel on Possibility: Modality Perfection and Dialectics	Nahum Brown	2020	Nonfictional
244	Historical Dictionary of Romantic Art and Architecture	Allison Lee Palmer	2019	Nonfictional
245	How Not to Be Wrong: The Power of Mathematical Thinking	Jordan Ellenberg	2014	Nonfictional
246	How Sexual Desire Works: The Enigmatic Urge	Frederick Toates	2014	Nonfictional
247	Human Nature	David Berlinski	2019	Nonfictional
248	Indian Philosophy: A Reader	Jonardon Ganeri	2020	Nonfictional
249	Lessons from Nanoelectronics: A New Perspective on Transport	Supriyo Datta	2012	Nonfictional
250	Leviathan or The Whale	Philip Hoare	2009	Nonfictional
251	Life Unfolding: How the Human Body Creates Itself	Jamie A. Davies	2014	Nonfictional
252	Liquid: The Delightful and Dangerous Substances That Flow Through Our Lives	Mark Miodownik	2018	Nonfictional
253	Marine Insurance Legislation	Robert Merkin, Jennifer Lavelle	2010	Nonfictional
254	Molecules Microbes and Meals: The Surprising Science of Food	Alan Kelly	2019	Nonfictional
255	Natural Law and Human Rights: Toward a Recovery of Practical Reason	Pierre Manent, Ralph C. Hancock, Daniel J. Mahoney	2020	Nonfictional
256	Night School: Wake Up to the Power of Sleep	Richard Wiseman	2014	Nonfictional
257	On Food and Cooking: The Science and Lore of the Kitchen rev. and updated	Harold McGee	2004	Nonfictional
258	Particle Physics: An Introduction	Robert Purdy	2018	Nonfictional
259	Philosophy: A Christian Introduction	James K. Dew, Jr.: Paul M. Gould	2019	Nonfictional
260	Preserving: The canning and freezing guide for all seasons	Pat Crocker	2012	Nonfictional
261	Principles of International Economic Law	Matthias Herdegen	2016	Nonfictional
262	Psychology moment by moment: a guide to enhancing your clinical practice with mindfulness and meditation	Elise E. Labbe	2011	Nonfictional
263	Radically Open Dialectical Behavior Therapy: Theory and Practice for Treating Disorders of Overcontrol	Thomas R. Lynch	2018	Nonfictional
264	Rest Play Grow: Making Sense of Preschoolers (or Anyone Who Acts Like One)	Deborah MacNamara	2016	Nonfictional
265	Revolution in Mind: The Creation of Psychoanalysis	George Makari	2008	Nonfictional
266	Same Sex Parenting Research: A Critical Assessment	Walter R. Schumm	2018	Nonfictional
267	Say What You Mean: A Mindful Approach to Nonviolent Communication	Oren Jay Sofer	2018	Nonfictional
268	Science in Black and White: How Biology and Environment Shape Our Racial Divide	Alondra Oubre	2020	Nonfictional
269	Secrets of Your Cells: Discovering your body s Inner Intelligence	Sondra Barrett	2013	Nonfictional
270	Sex and the Failed Absolute	Slavoj Zizek	2019	Nonfictional
271	Sex on Earth: A Celebration of Animal Reproduction	Jules Howard	2015	Nonfictional
272	Ship Registration: Law and Practice	Richard Coles, Edward Watt	2002	Nonfictional
273	Smells: A Cultural History of Odours in Early Modern Times	Robert Muchembled	2020	Nonfictional
274	Social Problems: Community Policy and Social Action	Anna Leon Guerrero	2018	Nonfictional
275	Social Psychology	Thomas E. Heinzen, Wind Goodfriend	2018	Nonfictional
276	Spheres of Influence: The Social Ecology of Racial and Class Inequality	Douglas S. Massey	2014	Nonfictional
277	Structural Geology	Haakon Fossen	2016	Nonfictional
278	Sustainable Landscape Construction: A Guide to Green Building Outdoors	Kim Sorvig J. William Thompson	2018	Nonfictional
279	The Age of Surveillance Capitalism: The Fight for a Human Future at the New Frontier of Power	Shoshana Zuboff	2019	Nonfictional
280	The Aquaponic Farmer: A Complete Guide to Building and Operating a Commercial Aquaponic System	Adrian Southern, Whelm King	2017	Nonfictional
281	The Architecture of Learning: Designing Instruction for the Learning Brain	Kevin D. Washburn	2010	Nonfictional

Continued on next page

Table S1 – *Continued from previous page*

	**Title**	**Author(s)**	**Year of Publication**	**Category**
282	The Battery: How Portable Power Sparked a Technological Revolution	Henry Schlesinger	2010	Nonfictional
283	The Charisma Myth: How Anyone Can Master the Art and Science of Personal Magnetism	Olivia Fox Cabane	2012	Nonfictional
284	The Chemistry of Wine: From Blossom to Beverage and Beyond	David R. Dalton	2017	Nonfictional
285	The Constitution in Exile: How the Federal Government Has Seized Power by Rewriting the Supreme Law of the Land	Andrew P. Napolitano	2007	Nonfictional
286	The Constitution: An Introduction	Michael Stokes Paulsen, Luke Paulsen	2015	Nonfictional
287	The Copenhagen Conspiracy	David Ferry	2019	Nonfictional
288	The dolphin in the mirror : exploring dolphin minds and saving dolphin lives	Reiss Diana	2011	Nonfictional
289	The Dream Universe: How Fundamental Physics Lost Its Way	David Lindley	2020	Nonfictional
290	The Empathy Advantage: Coaching Children to Be Kind Respectful and Successful	Lynne Azarchi	2021	Nonfictional
291	The End of Ownership: Personal property in the digital economy	Aaron Perzanowski, Jason Schultz	2016	Nonfictional
292	The Equations World	Boris Pritsker	2019	Nonfictional
293	The Evolution of Beauty: How Darwin s Forgotten Theory of Mate Choice Shapes the Animal World and Us	Richard O. Prum	2017	Nonfictional
294	The Greatest Show on Earth: The Evidence for Evolution	Richard Dawkins	2009	Nonfictional
295	The Kingdom of Infinite Number: A Field Guide	Bryan Bunch	2011	Nonfictional
296	The Law of State Immunity	Hazel Fox, QC and Philippa Webb	2013	Nonfictional
297	The Life Organic: The Theoretical Biology Club and the Roots of Epigenetics	Erik L. Peterson	2017	Nonfictional
298	The Limits of Epistemology	Markus Gabriel, Alex Englander	2020	Nonfictional
299	The Longing for Less: Living with Minimalism	Kyle Chayka	2020	Nonfictional
300	The Map That Changed the World: William Smith and the Birth of Modern Geology	Simon Winchester	2009	Nonfictional
301	The Master and His Emissary: The Divided Brain and the Making of the Western World	Iain McGilchrist	2019	Nonfictional
302	The Music of the Primes: Searching to Solve the Greatest Mystery in Mathematics	Marcus du Sautoy	2012	Nonfictional
303	The Narrow Corridor: States Societies and the Fate of Liberty	Daron Acemoglu, James A. Robinson	2019	Nonfictional
304	The New Evil: Understanding the Emergence of Modern Violent Crime	Michael H. Stone: Gary Brucato	2019	Nonfictional
305	The Origins of Political Order: From Prehuman Times to the French Revolution	Francis Fukuyama	2011	Nonfictional
306	The paradox of choice: why more is less	Barry Schwartz	2005	Nonfictional
307	The Pope of Physics: Enrico Fermi and the Birth of the Atomic Age	Gino Segrè Bettina Hoerlin	2016	Nonfictional
308	The Post Traumatic Stress Disorder Sourcebook: A Guide to Healing Recovery and Growth	Glenn R. Schiraldi	2016	Nonfictional
309	The Science of Communicating Science: The Ultimate Guide	Craig Cormick	2020	Nonfictional
310	The Silencing: How the Left is Killing Free Speech	Kirsten Powers	2015	Nonfictional
311	The Sixth Extinction: An Unnatural History	Elizabeth Kolbert	2014	Nonfictional
312	The Social Lens: An Invitation to Social and Sociological Theory	Kenneth Allan	2013	Nonfictional
313	The Story of Light	Ben Bova	2012	Nonfictional
314	The World According to Physics	Jim Al Khalili	2020	Nonfictional
315	Thinking fast and slow	Kahneman Daniel	2015	Nonfictional
316	This Life: Secular Faith and Spiritual Freedom	Martin Hagglund	2019	Nonfictional
317	Unconditional Parenting: Moving from Rewards and Punishments to Love and Reason	Alfie Kohn	2006	Nonfictional
318	Underground: A Human History of the Worlds Beneath Our Feet	Will Hunt	2019	Nonfictional

Continued on next page

Table S1 – *Continued from previous page*

	Title	Author(s)	Year of Publication	Category
319	Unequal Childhoods: Class Race and Family Life Second Edition with an Update a Decade Later	Annette Lareau	2011	Nonfictional
320	Unpeople: Britain s Secret Human Rights Abuses	Mark Curtis	2004	Nonfictional
321	What the Nose Tells the Mind	A. S. Barwich	2020	Nonfictional
322	Why Diets Make Us Fat: The Unintended Consequences of Our Obsession With Weight Loss	Sandra Aamodt	2016	Nonfictional
323	Why You Hear What You Hear: An Experiential Approach to Sound Music and Psychoacoustics	Eric J. Heller	2013	Nonfictional
324	Witcraft: The Invention of Philosophy in English	Jonathan Ree	2019	Nonfictional
325	Words That Change Minds: The 14 Patterns for Mastering the Language of Influence	Shelle Rose Charvet	2019	Nonfictional

Table S2: Median values of Approximate Entropy (ApEn) for all text properties and for all fictional text categories. ApEn values were analyzed for contemporary fictional (N = 204) vs. non-fictional (N = 122) texts, and for earlier fictional (N =206) vs. earlier non-fictional (N = 185) texts. The asterisks indicate whether the differences between the two text categories in contemporary or earlier text categories are statistically significant (Mann-Whitney U test; ns, not significant; *, $p \leq 0.05$; **, $p \leq 0.01$; and ***, $p \leq 0.001$). Values that are significantly higher within a pair of columns are shown in boldface. 95% confidence intervals for the median (according to [18]) are shown in parentheses. Data for earlier texts are from the study by Mohseni *et al.* [19].

	Contemporary		Earlier	
Text Property	Fictional	Non-Fictional	Fictional	Non-Fictional
Sentence Length	2.01 (1.99, 2.02)	1.98 (1.96, 2.00)^{ns}	1.87 (1.86, 1.88)	1.90 (1.88, 1.92)^{ns}
Noun	1.89 (1.88, 1.90)	1.90 (1.88, 1.91)^{ns}	**1.85 (1.84, 1.86)**	1.82 (1.81, 1.84)**
Verb	1.72 (1.71, 1.73)	**1.74 (1.73, 1.75)*****	1.714 (1.706, 1.723)	**1.756 (1.745, 1.764)*****
Adjective	1.38 (1.37, 1.39)	**1.63 (1.61, 1.64)*****	1.488 (1.469, 1.494)	**1.58 (1.55, 1.60)*****
Adverb	**1.51 (1.495, 1.516)**	1.38 (1.35, 1.40)***	**1.49 (1.48, 1.50)**	1.36 (1.34, 1.39)***
Pronoun	**1.72 (1.71, 1.73)**	1.27 (1.19, 1.32)***	**1.695 (1.685, 1.704)**	1.31 (1.28, 1.36)***
Preposition	1.62 (1.61, 1.63)	**1.66 (1.65, 1.65)*****	1.678 (1.672, 1.683)	**1.691 (1.686, 1.697)*****

Table S3: Median values of Shannon Entropy (ShEn) for all text properties and for all fictional text categories. ShEn values were analyzed for contemporary fictional (N = 204) vs. non-fictional (N = 122) texts, and for earlier fictional (N =206) vs. earlier non-fictional (N = 185) texts. The asterisks indicate whether the differences between the two text categories in contemporary or earlier text categories are statistically significant (Mann-Whitney U test; ns, not significant; *, $p \leq 0.05$; **, $p \leq 0.01$; and ***, $p \leq 0.001$). Values that are significantly higher within a pair of columns are shown in boldface. 95% confidence intervals for the median (according to [18]) are shown in parentheses. Data for earlier texts are from the study by Mohseni *et al.* [19].

	Contemporary		Earlier	
Text Property	Fictional	Non-Fictional	Fictional	Non-Fictional
Sentence Length	3.39 (3.37, 3.42)	**3.89 (3.84, 3.91)*****	3.96 (3.91, 4.03)	**4.10 (4.07, 4.16)*****
Noun	2.05 (2.02, 2.06)	**2.07 (2.05, 2.08)****	1.98 (1.97, 1.99)	1.97 (1.95, 1.99)^{ns}
Verb	1.79 (1.78, 1.80)	**1.82 (1.81, 1.83)*****	1.785 (1.779, 1.792)	**1.844 (1.836, 1.853)*****
Adjective	1.41 (1.40, 1.42)	**1.68 (1.66, 1.69)*****	1.52 (1.51, 1.53)	**1.63 (1.61, 1.66)*****
Adverb	**1.54 (1.52, 1.56)**	1.41 (1.38, 1.43)***	**1.52 (1.51, 1.53)**	1.40 (1.37, 1.42)***
Pronoun	**1.81 (1.807, 1.820)**	1.33 (1.24, 1.40)***	**1.79 (1.78, 1.80)**	1.37 (1.33, 1.42)***
Preposition	1.66 (1.657, 1.673)	**1.71 (1.705, 1.720)*****	1.736 (1.729, 1.744)	**1.76 (1.75, 1.77)*****

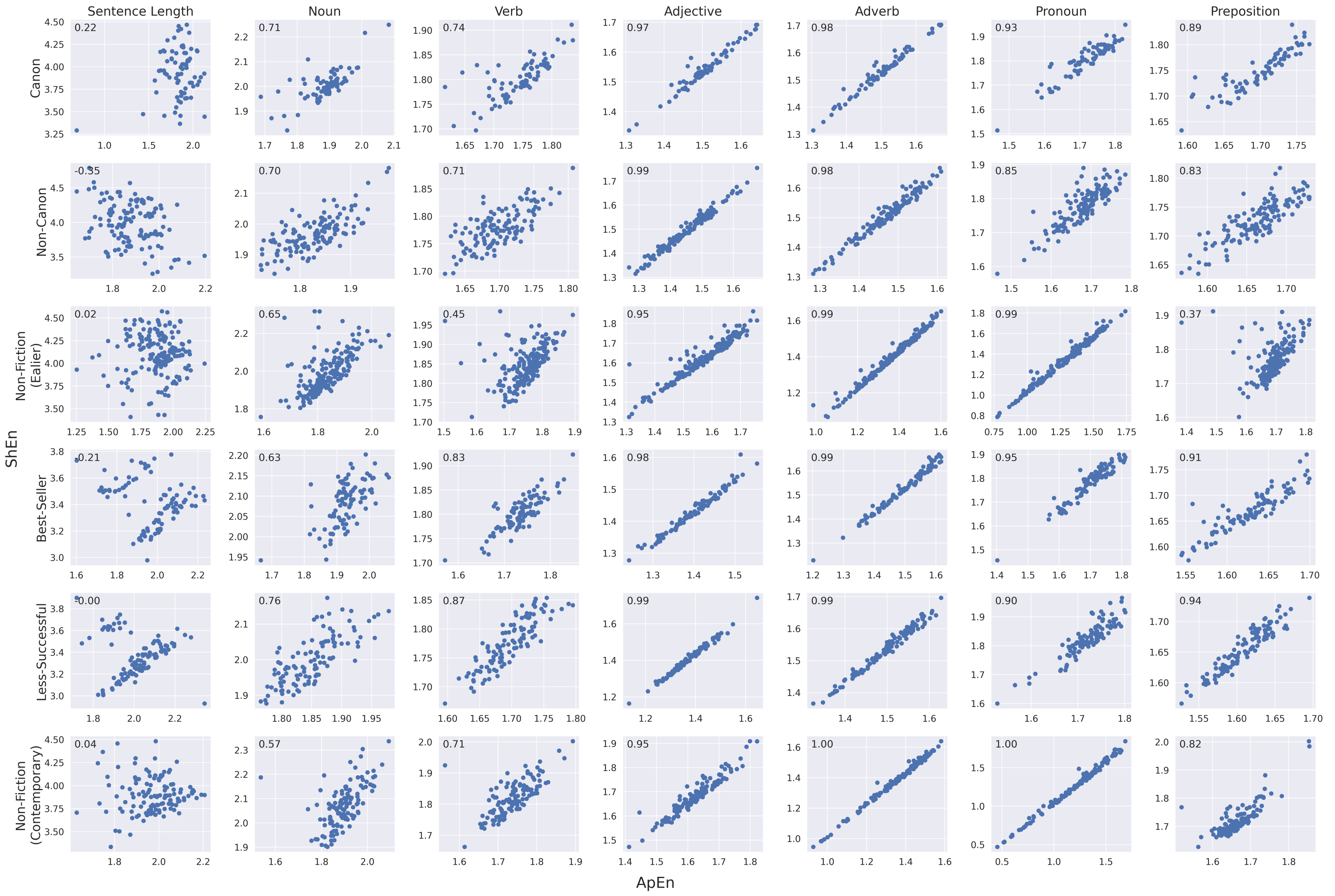

Figure S3. Approximate Entropy (ApEn; x-axis) vs. Shannon Entropy (ShEn; y-axis) for each text category (rows) and for each text property (columns). The correlation value is shown on the top left corner of each plot. To make the plots more readable, two outliers, which were both nonfictional texts written in the 19th century, were removed: A Dictionary of English Synonyms and Synonymous Expressions by Richard Soule and A Synopsis of the Birds of North America by John James Audubon.

References

1. Kumar, A.; Lease, M.; Baldridge, J. Supervised Language Modeling for Temporal Resolution of Texts. Proceedings of the 20th ACM International Conference on Information and Knowledge Management; Association for Computing Machinery: New York, NY, USA, 2011; CIKM '11, p. 2069–2072. doi:10.1145/2063576.2063892.
2. Garcia-Fernandez, A.; Ligozat, A.L.; Dinarelli, M.; Bernhard, D. When Was It Written? Automatically Determining Publication Dates. Proceedings of the 18th International Conference on String Processing and Information Retrieval; Springer-Verlag: Berlin, Heidelberg, 2011; SPIRE'11, p. 221–236.
3. Ciobanu, A.M.; Dinu, L.P.; Şulea, O.M.; Dinu, A.; Niculae, V. Temporal Text Classification for Romanian Novels set in the Past. Proceedings of the International Conference Recent Advances in Natural Language Processing RANLP 2013; INCOMA Ltd. Shoumen, BULGARIA: Hissar, Bulgaria, 2013; pp. 136–140.
4. Štajner, S.; Zampieri, M. Stylistic Changes for Temporal Text Classification. Lecture Notes in Computer Science. Springer, Berlin, Heidelberg, 2013, Vol. 8082, pp. 519–526. doi:10.1007/978-3-642-40585-3_65.
5. Gómez-Adorno, H.; Posadas-Duran, J.P.; Ríos-Toledo, G.; Sidorov, G.; Sierra, G. Stylometry-Based Approach for Detecting Writing Style Changes in Literary Texts. *Computación y Sistemas* **2018**, *22*, 47–53.
6. Efremova, J.; García, A.M.; Zhang, J.; Calders, T. Effects of Evolutionary Linguistics in Text Classification. International Conference on Statistical Language and Speech Processing. Springer, 2015, pp. 50–61. doi:10.46298/jdmdh.5864.
7. Liebeskind, C.; Liebeskind, S. Deep Learning for Period Classification of Historical Hebrew Texts. *Journal of Data Mining & Digital Humanities* **2020**, *2020*.
8. Gopidi, A.; Alam, A. Computational Analysis of the Historical Changes in Poetry and Prose. Proceedings of the 1st International Workshop on Computational Approaches to Historical Language Change; Association for Computational Linguistics: Florence, Italy, 2019; pp. 14–22. doi:10.18653/v1/W19-4702.
9. Lagutina, K.; Poletaev, A.; Lagutina, N.; Boychuk, E.; Paramonov, I. Automatic Extraction of Rhythm Figures and Analysis of Their Dynamics in Prose of 19th-21st Centuries. 26th Conference of Open Innovations Association (FRUCT), 2020, pp. 247–255. doi:10.23919/FRUCT48808.2020.9087430.
10. Lagutina, K.V.; Manakhova, A.M. Automated Search and Analysis of the Stylometric Features That Describe the Style of the Prose of 19th–21st Centuries. *Automatic Control and Computer Sciences* **2021**, *55*, 866–876. doi:10.3103/S0146411621070257.
11. Degaetano-Ortlieb, S. Stylistic Variation Over 200 Years of Court Proceedings According to Gender and Social Class. Proceedings of the Second Workshop on Stylistic Variation; Association for Computational Linguistics: New Orleans, 2018; pp. 1–10. doi:10.18653/v1/W18-1601.
12. Fankhauser, P.; Knappen, J.; Teich, E. Topical Diversification Over Time In The Royal Society Corpus; Jagiellonian University; Pedagogical University: Kraków, 2016; Digital Humanities.
13. Bizzoni, Y.; Degaetano-Ortlieb, S.; Fankhauser, P.; Teich, E. Linguistic Variation and Change in 250 Years of English Scientific Writing: A Data-Driven Approach. *Frontiers in Artificial Intelligence* **2020**, *3*. doi:10.3389/frai.2020.00073.
14. Wang, G.; Wang, H.; Sun, X.; Nan, W.; Wang, L. Linguistic complexity in scientific writing: A large-scale diachronic study from 1821 to 1920. *Scientometrics* **2022**, *128*, 441–460. doi:10.1007/s11192-022-04550-z.
15. Degaetano-Ortlieb, S.; Strötgen, J. Diachronic Variation of Temporal Expressions in Scientific Writing Through the Lens of Relative Entropy. Language Technologies for the Challenges of the Digital Age; Rehm, G.; Declerck, T., Eds.; Springer International Publishing: Cham, 2018; pp. 259–275.
16. Krielke, M.P.; Fischer, S.; Degaetano-Ortlieb, S.; Teich, E. System and use of wh-relativizers in 200 years of English scientific writing. Proceedings of the 10th International Corpus Linguistics Conference, Cardiff, Wales, UK, 2019.
17. US Novel Corpus. https://textual-optics-lab.uchicago.edu/us_novel_corpus. Accessed: 01-03-2023.
18. Zar, J.H. *Biostatistical Analysis*, 5 ed.; Pearson: Upper Saddle River, NJ, 2010.
19. Mohseni, M.; Redies, C.; Gast, V. Approximate Entropy in Canonical and Non-Canonical Fiction. *Entropy* **2022**, *24*. doi:10.3390/e24020278.
